# Federal Payments for Coronary Revascularization Procedures Among Dual Enrollees in Medicare Advantage and the Veterans Affairs Health Care System

**DOI:** 10.1001/jamanetworkopen.2020.1451

**Published:** 2020-04-06

**Authors:** Elias J. Dayoub, Elena L. Medvedeva, Sameed Ahmed M. Khatana, Ashwin S. Nathan, Andrew J. Epstein, Peter W. Groeneveld

**Affiliations:** 1 Corporal Michael J. Crescenz Veterans Affairs Medical Center, Philadelphia, Pennsylvania; 2 Center for Cardiovascular Outcomes, Quality, and Evaluative Research, University of Pennsylvania, Philadelphia; 3 Leonard Davis Institute of Health Economics, University of Pennsylvania, Philadelphia; 4 Division of Cardiovascular Medicine, Hospital of the University of Pennsylvania, Philadelphia

## Abstract

**Importance:**

More than 1 million US veterans are dually enrolled in a Medicare Advantage (MA) plan and in the Veterans Affairs (VA) health care system. The federal government prepays private MA plans to cover veterans; if a dually enrolled veteran receives an MA-covered service at the VA, the government is making 2 payments for the same service. It is not clear what proportion of veterans dually enrolled in VA and MA are undergoing coronary revascularization at VA vs non-VA hospitals.

**Objective:**

To describe where veterans who are enrolled in both VA and MA undergo coronary revascularization and the associated costs.

**Design, Settings, and Participants:**

This is a cohort study consisting of US veterans dually enrolled in VA and MA from January 1, 2010, to December 31, 2013, who had at least 1 VA encounter and underwent coronary revascularization during the study period. Data were analyzed from April 2019 to September 2019.

**Main Outcomes and Measures:**

Number of coronary artery bypass graft (CABG) operations and percutaneous coronary interventions (PCIs) performed through the VA and through MA during years 2010 to 2013, and the associated VA costs of coronary revascularization. In addition, multivariable logistic regression was performed to assess patient factors associated with receiving care through the VA.

**Results:**

A total of 18 874 VA users with concurrent MA enrollment who underwent coronary revascularization during 2010 to 2013 were identified (mean [SD] age, 75.3 [8.8] years; 18 739 men [99.0%]). Enrollees were predominantly white (17 457 patients [92.0%]). Among patients, 4115 (22.0%) underwent either CABG or PCI through the VA only, 14 281 (75.0%) did so through MA only, and 478 (2.5%) underwent coronary revascularization procedures through both payers. From 2010 to 2013, these veterans underwent 4764 coronary revascularization procedures (721 CABGs and 3043 PCIs) that cost the VA $214.7 million ($115.8 million for CABGs and $99.0 million for PCIs). In multivariable analysis, nonwhite patients were more likely than white patients to undergo coronary revascularization through the VA (odds ratio, 1.73; 95% CI, 1.52-1.96; *P* < .001), and for each year of age, veterans were less likely to undergo coronary revascularization through the VA (odds ratio, 0.95; 95% CI, 0.94-0.95; *P* < .001). There was no statistically significant association between undergoing coronary vascularization through the VA and distance in miles to the nearest VA hospital (odds ratio, 1.00; 95% CI, 0.99-1.00; *P* = .30).

**Conclusions and Relevance:**

A substantial share of VA users concurrently enrolled in an MA plan underwent coronary revascularization procedures through the VA, incurring significant duplicative federal health care spending. Given the financial pressures facing both Medicare and the VA, government officials should consider policy solutions to mitigate redundant spending.

## Key Points

**Question:**

Where do US veterans who are enrolled in both the Veterans Affairs (VA) health care system and Medicare Advantage undergo coronary revascularization, and what are the associated charges?

**Findings:**

In a national cohort study, among the 18 874 dually enrolled veterans who underwent coronary revascularization from 2010 to 2013, 4115 underwent a procedure through the VA system only, and 478 did so through both the VA and Medicare Advantage. These coronary revascularizations cost the VA $214.7 million, reflecting duplicative spending, because the federal government prepays private Medicare Advantage plans to cover veterans.

**Meaning:**

These findings suggest that US government officials should consider policy solutions to mitigate duplicative federal spending, given the financial pressures facing both Medicare and the VA.

## Teaser

This cohort study examines where US veterans who are enrolled in both the Veterans Affairs (VA) health care system and Medicare Advantage undergo coronary revascularization and the associated costs.

## Introduction

Approximately 1.2 million veterans are dually enrolled in 2 federally funded health care systems: the Medicare Advantage (MA) program, which is administered by the Centers for Medicare & Medicaid Services, and the Veterans Affairs (VA) health care system, which is administered by the Veterans Health Administration in the US Department of Veterans Affairs.^1^ The federal government prepays a capitation to private MA plans to provide comprehensive care to enrollees, and, in turn, the MA plan is responsible for paying any costs associated with services covered by Medicare. However, whenever a patient who is dually enrolled in both MA and VA receives a Medicare-covered service through the VA, the government has made 2 payments for the same service.^2,3,4^ A previous study^2^ found that the VA annual health care cost for MA enrollees was $3.2 billion in 2009.

Moreover, Medicare plans are prohibited by law from making payments for services already paid for by another government entity. Thus, the VA cannot collect reimbursements from MA plans for Medicare-covered services, whereas the VA does bill private insurers when providing care to veterans enrolled in a non-Medicare private insurance plan.

Ischemic heart disease is highly prevalent in the veteran population.^5^ The use of costly cardiovascular procedures, such as coronary revascularization, by veterans dually enrolled in MA and VA has not been well characterized. Using a national cohort of veterans who use the VA and are concurrently enrolled in MA, we examined the receipt of coronary revascularization services in the VA and MA, the costs incurred by the VA to provide coronary revascularization to MA enrollees, and patient characteristics associated with undergoing revascularization at the VA vs through MA.

## Methods

The institutional review board of the Corporal Michael J. Crescenz Veterans Affairs Medical Center approved the study protocol. Because it was infeasible to obtain informed consent from nearly 200 000 veterans and because the data were deidentified, a waiver was granted by the institutional review board. The study follows the Strengthening the Reporting of Observational Studies in Epidemiology (STROBE) reporting guideline.^6^

### Data Sources

We obtained administrative health care data from January 1, 2010, through December 31, 2013, from the VA’s Corporate Data Warehouse, which contains detailed information on all inpatient, outpatient, laboratory, and pharmacy encounters through the VA health care system. To determine use of services in MA plans, we linked patient-level records from the Medicare Healthcare Effectiveness Data and Information Set (HEDIS) data, which include information on the use of acute medical and surgical inpatient care and ambulatory encounters. All MA plans are required to submit HEDIS data to Centers for Medicare & Medicaid Services.

### Cohort Selection

Our study cohort included all veterans enrolled in MA and with at least 1 clinical encounter in the VA health care system from January 1, 2010, to December 31, 2013, and who underwent percutaneous coronary intervention (PCI) or coronary artery bypass graft (CABG) surgery during the same period. We examined all administrative claims in the 12 months preceding revascularization to identify comorbidities and antecedent health events.

For MA coverage, HEDIS reports at the individual level whether a patient is enrolled in an MA plan in a given calendar year. For each year from 2010 to 2013, we considered a patient to be enrolled in MA if they had continuous enrollment in a MA plan for the entire calendar year. Patients were considered enrolled in the VA once they began receiving VA services and were included in a calendar year if it began before or during the first calendar quarter (ie, January 1 to March 31) of that year. Thus, patients were considered coenrolled in MA and VA when they were covered by both payers in a given calendar year.

### Outcomes

The primary outcome was the total number of coronary revascularization procedures (ie, PCI and CABG) performed through either the VA or MA, by year, from 2010 to 2013. In the VA data, PCI and CABG were identified in administrative claims using *International Classification of Diseases, Ninth Revision* procedure codes (ie, 00.66, 36.06, 36.07, and 36.09 for PCI and 36.1x for CABG)^7,8,9,10^ and diagnosis-related group codes (ie, 246-249 for PCI and 231-236 for CABG).^11^ We further identified procedures in the VA data that were performed within the VA system or were performed through fee-basis care. Fee-basis care includes services financed by the VA for care provided outside the VA health care system when VA care is not available or is unable to provide the service within the VA. Any CABG and PCI procedures performed for veterans and covered by MA were identified in the HEDIS data, which reports these procedures at the individual level by year.

With the number of coronary revascularization procedures performed through both VA and MA, we also assessed VA medical center (VAMC) variation in the proportion of procedures performed through the VA. Within each calendar quarter, veterans in this cohort were assigned to a primary VAMC, defined as the VAMC where the veteran had received the plurality of his or her VA health care, quantified as inpatient hospital days plus outpatient clinic visits. The proportion of coronary revascularization procedures performed through the VA was calculated by the number of procedures performed through a VAMC compared with the number of procedures performed through MA among veterans assigned to the same VAMC.

Once the numbers of cardiovascular revascularization procedures were identified, we calculated the costs incurred by the VA to provide coronary revascularization. Because the VA is funded through Congressional appropriation, the VA does not assign costs or charges for individual services. Instead, the VA Health Economics Resource Center uses average cost methods to estimate the costs of individual services provided by the VA.^12^ For fee-basis care, we used the actual payments made by the VA to finance PCI and CABG occurring outside the VA system. The costs were inclusive of inpatient and outpatient services received from 30 days before to 90 days after the date the coronary revascularization procedure was performed.

Finally, we assessed patient-level factors associated with the payer providing coronary revascularization (ie, VA vs MA). Using a logistic regression model with receipt of PCI or CABG through the VA vs through MA as the dependent variable, the independent variables measured were age in years, sex, race (ie, white and nonwhite), US Census division (ie, New England, Middle Atlantic, East North Central, West North Central, South Atlantic, East South Central, West South Central, Mountain, and Pacific), National Center for Health Statistics urban-rural classification (ie, grouped into 3 categories: large fringe and large central metropolitan counties, small and medium metropolitan counties, and noncore and micropolitan counties),^13^ distance in miles to the nearest VA hospital, and Elixhauser Comorbidity Index score.^14^ Patients who had coronary revascularization performed through both MA and VA were excluded from the logistic regression model.

### Statistical Analysis

Differences in baseline characteristics between groups were compared using χ^2^ tests for categorical variables and analysis of variance for continuous variables. All statistical tests were 2-sided, with *P* < .05 indicating statistical significance. In the logistic regression model, estimated adjusted odds ratios (ORs) are reported with 95% CIs. All analyses were conducted using SAS statistical software version 9.4 (SAS Institute). Data were analyzed from April 2019 to September 2019.

## Results

Of the 18 874 VA users with concurrent MA enrollment who underwent coronary revascularization during 2010 through 2013, 18 739 (99.0%) were men, 17 457 (92.0%) were white, and the mean (SD) age was 75.3 (8.8) years. Among these patients, 14 281 (75.0%) underwent either CABG or PCI through MA only, 4115 (22.0%) did so through the VA only, and 478 (2.5%) underwent coronary revascularization through both (Table 1). In unadjusted analysis, veterans who received care through MA were older (age ≥80 years, 4433 patients [31.0%] vs 762 patients [15.8%]) and more likely to be white (13 418 patients [94.0%] vs 3606 patients [87.6%]) compared with those who received care through the VA. Although patients who underwent coronary revascularization at the VA disproportionately lived in more-urban counties compared with those who received care through MA (large central metropolitan county, 1294 patients [31.4%] vs 3275 patients [22.9%]), there was no statistically significant difference in the distance to a VA hospital between patients who underwent coronary revascularization through the VA or through MA (mean [SD], 14.3 [13.1] miles [23.0 {21.1} km] vs 14.7 [13.7] miles [23.7 {22.0} km]; difference, 0.4 mile [0.6 km]; 95% CI, −0.1 to 0.9 mile [−0.2 to 1.4 km]; *P* = .53).

**Table 1.** Demographic Characteristics of Veterans Enrolled in the VA Health Care System and MA Who Underwent Coronary Revascularization, by Payer

Characteristic	Patients, No. (%)			*P* value^a^
	VA (n = 4115)	MA (n = 14 281)	VA and MA (n = 478)	
Age, y				
<65	830 (20.2)	1434 (10.0)	132 (27.6)	<.001
65-69	1120 (27.2)	2220 (15.5)	126 (26.4)	
70-74	751 (18.3)	2565 (18.0)	70 (14.6)	
75-79	762 (18.5)	3629 (25.4)	91 (19.0)	
≥80	762 (15.8)	4433 (31.0)	59 (12.3)	
Male	4073 (99.0)	14 197 (99.4)	469 (98.1)	.14
Race				
Nonwhite	506 (12.3)	855 (6.0)	44 (9.2)	<.001
White	3606 (87.6)	13 418 (94.0)	433 (90.6)	
Unknown	3 (0.1)	8 (0.1)	1 (0.2)	
US Census division				
New England	106 (2.6)	524 (3.7)	16 (3.3)	<.001
Middle Atlantic	328 (8.0)	2850 (20.0)	32 (6.7)	
East North Central	530 (12.9)	2349 (16.4)	67 (14.0)	
West North Central	459 (11.2)	1299 (9.1)	49 (10.3)	
South Atlantic	924 (22.5)	2884 (20.2)	119 (24.9)	
East South Central	322 (7.8)	762 (5.3)	33 (6.9)	
West South Central	521 (12.7)	1420 (9.9)	61 (12.8)	
Mountain	393 (9.6)	955 (6.7)	60 (12.6)	
Pacific	384 (9.3)	1015 (7.1)	34 (7.1)	
Unknown	148 (3.6)	223 (1.6)	7 (1.5)	
Urban-rural county classification				
Large central metropolitan county	1294 (31.4)	3275 (22.9)	119 (24.9)	<.001
Large fringe metropolitan county	806 (19.6)	3473 (24.3)	84 (17.6)	
Medium metropolitan county	968 (23.5)	3660 (25.6)	132 (27.6)	
Small metropolitan county	275 (6.7)	1645 (11.5)	57 (11.9)	
Micropolitan county	353 (8.6)	1241 (8.7)	43 (9.0)	
Noncore county	243 (5.9)	710 (5.0)	29 (6.1)	
Unknown	176 (4.3)	277 (1.9)	14 (2.9)	
Distance to nearest VA hospital, miles^b^				
<2	381 (9.3)	1081 (7.6)	48 (10.0)	<.001
2-5	900 (21.9)	3063 (21.4)	121 (25.3)	
6-10	768 (18.7)	3187 (22.3)	92 (19.2)	
11-15	541 (13.1)	2002 (14.0)	53 (11.1)	
16-20	386 (9.4)	1483 (10.4)	42 (8.8)	
21-25	253 (6.1)	1026 (7.2)	23 (4.8)	
26-50	622 (15.1)	1833 (12.8)	77 (16.1)	
>50	75 (1.8)	286 (2.0)	9 (1.9)	
Unknown	189 (4.6)	320 (2.2)	13 (2.7)	
Coronary revascularization procedure				
Coronary artery bypass graft surgery	1651 (40.1)	2857 (20.0)	137 (28.7)	<.001
Percutaneous coronary intervention	2523 (61.3)	11 786 (82.5)	369 (77.2)	

Abbreviations: MA, Medicare Advantage; VA, Veterans Affairs.

^a^ Differences in baseline characteristics between groups were compared using χ^2^ tests.

^b^ To convert miles to kilometers, multiply by 1.61.

### Coronary Revascularization Utilization and Costs of VA Services

**Figure 1.**
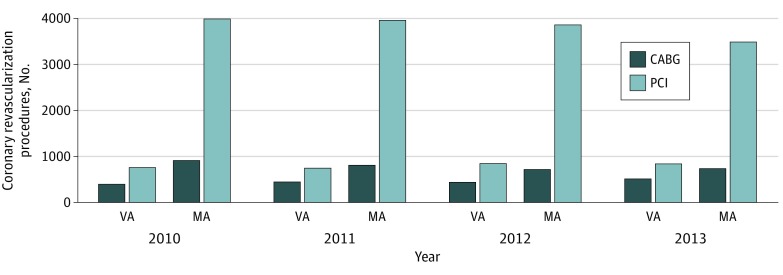
Coronary Revascularization Procedures Performed Through the Veterans Affairs (VA) Health Care System and Medicare Advantage (MA) for VA Users With Concurrent MA Enrollment Bars represent the number of coronary artery bypass graft (CABG) operations and percutaneous coronary interventions (PCIs) paid for by VA and MA for VA users with concurrent MA enrollment.

From 2010 to 2013, 4764 coronary revascularization procedures (1721 CABGs and 3043 PCIs) were covered by the VA (Figure 1). Of these procedures, 3958 (83.0%) were performed at a VA hospital and 806 (17.0%) were covered under fee-basis care. The number of coronary revascularization procedures performed through MA totaled 18 481 (3179 CABGs and 15 302 PCIs). In this cohort, the mean percentage of coronary revascularization procedures performed through the VA was 21.0%. When stratifying the cohort by VAMC primary assignment, the median VAMC provided 18.0% (interquartile range, 9.0%-32.0%) of coronary revascularization procedures through the VA.

**Figure 2.**
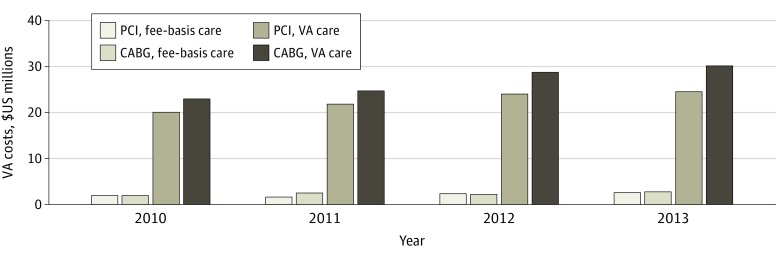
Estimated Costs to the Veterans Affairs (VA) for Coronary Revascularization Provided to Veterans With Concurrent Enrollment in Medicare Advantage, 2010 to 2013 Bars represent the costs incurred by the VA for coronary artery bypass graft (CABG) surgery and percutaneous coronary intervention (PCI) through direct care provided through the VA system and through fee-basis care.

From 2010 to 2013, the total expenditures of VA care for coronary revascularization in this sample were $214.7 million, with $115.8 million spent on CABG and $99.0 million spent on PCI (Figure 2). The annual amount spent on coronary revascularization care increased from $47.0 million in 2010 to $59.8 million to 2013. From 2010 to 2013, VA expenditures from fee-basis care totaled $17.7 million, or 8.2% of the total expenditures for coronary revascularization. Annual expenditures from fee-basis care increased from $3.9 million in 2010 to $5.2 million in 2013. The mean per-patient cost of a coronary revascularization procedure was $45 000: $67 000 for CABG and $33 000 for PCI. The estimated payment by the federal government to private MA plans to cover this cohort of veterans was $188 million annually.

### Factors Associated With Undergoing Coronary Revascularization Through VA

In multivariable analysis (Table 2), older patients were less likely to undergo coronary revascularization through the VA (OR, 0.95; 95% CI, 0.94-0.95; *P* < .001). Nonwhite patients were more likely than white patients to undergo coronary revascularization through the VA (OR, 1.73; 95% CI, 1.52-1.96; *P* < .001). Veterans residing in small and medium metropolitan counties were less likely to undergo coronary revascularization through the VA, compared with patients living in noncore and micropolitan counties (OR, 0.76; 95% CI, 0.68-0.86; *P* < .001). Patients with a higher Elixhauser Comorbidity Index score were less likely to undergo coronary revascularization through the VA (OR, 0.99; 95% CI, 0.98-0.99; *P* < .001). There was no statistically significant association between undergoing coronary vascularization through the VA and distance in miles to the nearest VA hospital (OR, 1.00; 95% CI, 0.99-1.00; *P* = .30).

**Table 2.** Multivariable Logistic Regression on Factors Associated With Receiving Coronary Revascularization Through the VA Health Care System

Characteristic	Undergoing coronary revascularization through VA	
	OR (95% CI)	*P* value
Age, y	0.95 (0.94-0.95)	<.001
Female	1.27 (0.85-1.89)	.24
Nonwhite race	1.73 (1.52-1.96)	<.001
US Census division		
Pacific	1 [Reference]	
New England	0.63 (0.49-0.81)	<.001
Middle Atlantic	0.31 (0.26-0.37)	<.001
East North Central	0.62 (0.53-0.73)	<.001
West North Central	1.01 (0.85-1.19)	.93
South Atlantic	0.82 (0.70-0.94)	.01
East South Central	0.96 (0.80-1.16)	.68
West South Central	0.85 (0.72-0.99)	.04
Mountain	1.17 (0.98-1.39)	.08
Urban-rural county classification		
Noncore or micropolitan county	1 [Reference]	
Small or medium metropolitan county	0.76 (0.68-0.86)	<.001
Large fringe or large central metropolitan county	1.10 (0.98-1.24)	.11
Distance to nearest VA hospital, miles	1.00 (0.99-1.00)	.30
Elixhauser Comorbidity Index score	0.99 (0.98-0.99)	<.001

Abbreviations: OR, odds ratio; VA, Veterans Affairs.

## Discussion

Among VA users with concurrent MA enrollment, we found a substantial proportion who underwent coronary revascularization through the VA health care system. Of the 18 874 dually enrolled patients who, during 2010 to 2013, used the VA at least once and underwent either CABG or PCI, 24.5% underwent coronary revascularization through the VA (22.0% through the VA only and 2.5% through both VA and MA). The total cost of duplicative federal spending to provide coronary revascularization through the VA for patients also enrolled in MA was $214.7 million from 2010 to 2013. We also found that among these patients, younger, nonwhite veterans living in urban and rural counties were more likely to undergo CABG or PCI through the VA, whereas distance to a VA hospital was not independently associated with the choice of VA vs MA for coronary revascularization.

A prior study^2^ examining resource utilization for all health care and the associated VA costs for patients dually enrolled in VA and MA found that 50% of these veterans used care through both the VA and MA, and that the VA financed 15% of all acute medical and surgical admissions. In our analysis of a high-cost population undergoing CABG or PCI, we found a higher proportion of coronary revascularization procedures financed by the VA. Moreover, the prior study^2^ found the mean per-patient VA costs to be $5700 in 2009, whereas we found the mean per-patient cost of a coronary revascularization procedure to be $45 000 ($67 000 for CABG and $33 000 for PCI) during our study period, reflecting the significant resources used to care for veterans requiring CABG or PCI.

The annual amount the federal government pays in capitation payments to private MA plans to cover an enrollee is calculated using a combination of the enrollee’s county of residence and risk adjustment using the health status of the beneficiary. The mean MA capitation payment in 2010 was $9970.^15^ The patients dually enrolled in MA and VA who underwent CABG or PCI would likely have had above-average capitation payments given risk adjustment. Nevertheless, using the mean MA capitation as a conservative approximation, the estimated payment by the federal government to private MA plans to cover this cohort of veterans was $188 million annually.

This magnitude of duplicate federal payments for covering medical care for veterans dually enrolled in MA and VA is likely to increase over time, given that enrollment in MA has steadily increased over the past decade and is expected to continue increasing.^16^ The number of enrollees in MA has grown from 8.4 million in 2007 to 19.0 million in 2017, an increase from 19% to 34% of all Medicare beneficiaries.^17^ One proposed policy approach to mitigate duplicate payments is to allow the VA to collect reimbursements from MA plans for Medicare-covered services provided by the VA, similar to how the VA bills private insurers when providing care to veterans enrolled in a non-Medicare private insurance plan.^2,18,19^ Section 1862 of the Social Security Act prohibits Medicare from making payment for services that are paid for directly or indirectly by another government entity. This Section was enacted in 1965, before the introduction of Medicare Advantage and, thus, may be outdated for the current environment, which includes private MA plans. Conversely, proposed policies should consider instances when MA plans provide substantial services to veterans and whether MA plans should analogously be permitted to collect reimbursements from the VA.

Another policy approach to help reduce duplicative expenditures for beneficiaries enrolled in both VA and MA is to adjust capitation payments to MA plans for veterans who receive a significant amount of care through the VA system. The US Government Accountability Office evaluated this issue and noted that Centers for Medicare & Medicaid Services lacks data on VA diagnosis and utilization and, thus, does not include it in their method for determining MA capitation payments.^20^ Their primary recommendation was for Centers for Medicare & Medicaid Services to obtain VA utilization data and use them to make additional adjustments to MA plan payments as appropriate.

We found that dually enrolled, nonwhite veterans were more likely to use VA services, consistent with past studies that have examined characteristics of veterans enrolled in both VA and MA and where they receive care.^21,22,23^ Unlike most past studies that examined veterans dually enrolled in fee-for-service Medicare, we did not find a negative association between distance to VA hospital and receipt of care at the VA.^22,24^ This finding may be associated with the health maintenance organization model of Medicare Advantage; a past study^25^ examining hospital choice for CABG found that patients with health maintenance organization insurance plans were less sensitive to hospital proximity. Moreover, a study^26^ examining MA plans in 20 US counties found that 1 in 5 plans included fewer than 5 cardiothoracic surgeons in their network of physicians.

### Limitations

This study has limitations inherent to retrospective studies using administrative claims. Our study data lacked detailed clinical information; however, we used validated administrative codes for CABG and PCI to optimally identify procedures with the data available. Because HEDIS does not provide detailed encounter-level data, diagnostic codes to calculate the Elixhauser Comorbidity Index score for patients came solely from VA data; thus, not all diagnostic codes may have been captured and Elixhauser Comorbidity Index scores may be underestimated. Second, outside of fee-basis care, the VA expenditures reported are estimated, given that the VA does not assign costs or charge for individual services. Nonetheless, the estimated costs were derived from an average cost method that is commonly used by the VA Health Economics Resource Center.^12^ In addition, we were unable to calculate the exact amount of capitation payments made to private MA plans from the federal government, because this information is not publicly available. We also were unable to assess the effect that coenrolled veterans who only underwent coronary revascularization through MA had on allocation of federal dollars through the Veterans Equitable Resource Allocation system and, thus, likely underestimated redundant federal spending. Furthermore, our selection criteria required patients to have at least 1 inpatient or outpatient administrative record in the VA health care system to be identified and included in the cohort. We, therefore, could not observe dually enrolled veterans who received care exclusively through MA. Because our primary objective was to describe duplicative federal spending, dually enrolled patients receiving care solely through MA would not affect these results.

## Conclusions

A substantial share of VA users with concurrent MA enrollment underwent coronary revascularization procedures through the VA, incurring duplicative federal health care spending of $214.7 million during 2010 to 2013. The growing number of Medicare beneficiaries opting into MA plans may be associated with an increase in duplicative federal spending for veterans dually enrolled in VA and MA. Given the financial pressures facing both Medicare and the VA, government officials should consider policy solutions to mitigate redundant spending.
